# Human papillomavirus infection and follow-up on positive results in 7222 female samples obtained from 2016 to 2019 in Hefei, China

**DOI:** 10.7717/peerj.10179

**Published:** 2020-10-20

**Authors:** Liduo Peng, Liping Yin, Yaqian Dai, Yuanjing Peng, Yuanhong Xu, Huaqing Hu, Jinping Qiao

**Affiliations:** 1 Department of Clinical Laboratory, The First Affiliated Hospital of Anhui Medical University, Hefei, Anhui, China; 2 Health Management Center, The First Affiliated Hospital of Anhui Medical University, Hefei, Anhui, China; 3 Department of Clinical Laboratory, Anqing Municipal Hospital, Anqing, Anhui, China

**Keywords:** Hefei, Human papillomavirus, HPV genotype assay, HPV vaccine, Cervical cancer

## Abstract

**Background:**

Human papillomavirus (HPV) infection rates in women vary regionally. This study analyzed HPV infection in women of different age groups in Hefei, China, performed follow-up on positive cases, and discussed infection prognoses.

**Methods:**

Samples (7,222) of exfoliated cervical cells were collected in Hefei and tested with an HPV assay kit against 27 HPV genotypes. Statistical software was used to analyze the data.

**Results:**

The total positive rate of infection was 17.13% (1,068 women), and the 51–60-year age group had the highest HPV infection rate (19.82%). There were statistically significant differences between rates in the 21–30 and 31–40 (*P* = 0.002), 21–30 and 41–50 (*P* = 0.0003), 21–30 and 51–60 (*P* = 0.00003), and 51–60 and >60 age groups (*P* = 0.046). High-risk infection (15.67%) and single infection (13.01%) were the main types of HPV infection. The dominant genotypes of high-risk infection were HPV 52 (2.42%), HPV 16 (2.01%), HPV 53 (1.43%), HPV 58 (1.32%) and HPV 66 (1.01%). We conducted follow-up on cases in 69 of 94 women who had a history of 1–4 years of positive infection, and in 18 (seven treated, 11 untreated) patients, infection status turned negative (26.09%). Seventeen of the fifty-one women whose infections did not turn negative received treatment. Persistent infection was predominantly observed in high-risk genotypes (56 of 69).

**Conclusions:**

The results recommend that women in Hefei improve health awareness and receive a 9-valent vaccine. Additionally, women with persistent infections should consult a gynecologist to prevent cervical lesions.

## Introduction

Papillomaviruses are a family of viruses that infect animals and humans. Almost 200 human papillomavirus (HPV) types have been identified, 40 of which colonize the reproductive tract (Bravo & Félez-Sánchez, 2015). They are divided according to their carcinogenic properties into high-risk types (HPV 16, 18, 31, 33, 35, 39, 45, 51, 52, 56, 58, 59, 68, 73, and 82), potentially high-risk types (HPV 26, 53, and 66) and low-risk types (HPV 6, 11, 40, 42, 43, 44, 54, 61, 70, 72, and 81) (Asiaf et al., 2014; Mariani et al., 2010).

HPV infections are mostly subclinical and transient. The median duration of infection is approximately 13 months for carcinogenic HPV types and 8 for non-carcinogenic types. Clearance time is also affected by HPV type: approximately 8 months for carcinogenic types and 5 months for non-carcinogenic types (Brianti, Flammineis & Mercuri, 2017). Although HPV can evade the host immune system and downgrade innate immunity, approximately 90% of women can naturally clear primary HPV infection within two years; only 1%–2% of infections develop into chronic cervical cancer. However, in some women with persistent high-risk HPV infections, the risk of infection developing into the precancerous state is increased significantly (Deligeoroglou et al., 2013; McBride, 2017). Numerous studies have confirmed that persistent infection with high-risk HPV is a major risk factor for cervical intraepithelial neoplasia (CIN), which may range from CIN1 to CIN3 and cancer (Chan et al., 2019; Kjaer et al., 2010; McCredie et al., 2008; Moscicki et al., 2010). HPV 16 and 18 cause 70% of cervical cancers and precancerous cervical lesions. Non-carcinogenic HPV types (especially 6 and 11) can cause condyloma acuminatum and papillomatosis of the respiratory tract (Harden & Munger, 2017; Vonsky et al., 2019).

The incidence of cervical cancer among young women in China showed an upward trend from 2000 to 2014 (Li et al., 2017). In 2012, 630,000 new cancer cases worldwide were attributed to HPV infection, accounting for 4.5% of all cancers, with cervical cancer accounting for 83% (Martel et al., 2017). There were 110,894 new cancer cases attributed to HPV in 2014; the top three cancer types were cervical cancer (99,253, 90%), anal cancer (3936, 4%), and head and neck cancer (3340, 3%) (Duan et al., 2020). Among cancers that endanger women’s health, cervical cancer ranks fourth in morbidity and mortality rate, with approximately 570,000 patients and 311,000 deaths worldwide in 2018 (Bray et al., 2018). In China, cervical cancer places a heavy burden on women’s health and social development. Despite the continuous development of relevant screening and prevention measures, the diagnostic incidence of cervical cancer has not been controlled effectively.

HPV DNA testing is currently a routine part of physical examinations for women. The US Preventive Services Task Force (USPSTF) recommended that women aged 30–65 years should undergo either cytology testing alone every 3 years or high-risk HPV types testing alone every 5 years to reduce cervical cancer incidence and mortality (USPSTF, 2017). This study analyzed the HPV genotype assay results of 7,222 female samples and conducted telephone follow-up of selected women to analyze the status and distribution of HPV infection in Hefei in order to provide a reference for the prevention and treatment of HPV infection.

## Materials & Methods

### Research participants

The samples (7,222) of exfoliated cervical cells for the HPV genotype assay were obtained from the Health Management Center of the First Affiliated Hospital of Anhui Medical University from January 1, 2016 to October 31, 2019. After analysis, it was found that some women had multiple HPV tests during this period. The samples were taken from 6,234 women with mean age of 43 ± 10 years who underwent physical examinations. We requested an exemption from informed consent from the Ethics Review Committee for research using medical records or biological specimens obtained in previous clinical diagnosis and treatment, and applied for exemption from informed consent. This study was approved by the Ethics Review Committee of the First Affiliated Hospital of Anhui Medical University, Anhui, China (reference number: Quick-PJ 2020-03032).

### Reagents and instruments

Shanghai Tellgen HPV assay kit against 27 genotypes along with sterile collector, cell preservation solution, DNA extraction reagent, amplification reagents, and hybridization reagents (Shanghai Tellgen Life Science Co., Ltd., Shanghai, China); LifeECO PCR platform (Hangzhou Bioer Technology Co., Ltd., Hangzhou, China); Luminex 200 analyzer (Luminex Corp., Austin, TX, USA); ThermoMixer C metal bath and 5424R centrifuge (Eppendorf, Hamburg, Germany).

### Collection, storage, and transport of specimens

A disposable, sterile collector was used to collect the extirpated cervical cells and put them into the cell preservation solution. Specimens were sent for inspection at room temperature within 2 h or stored at −20 °C (retention period 60 days).

### DNA extraction

The samples were shaken for 20 s, and 200 µL of the mixture was placed into a 1.5 mL tube. The tube was centrifuged at 15,000 rpm for 5 min, and the supernatant was removed. DNA extraction reagent (200 µL) was added to the tube and mixed well. The tube was incubated at 100 °C for 15 min in the a metal bath. The tube was then centrifuged at 15,000 rpm for 5 min, and the supernatant was transferred to a new tube for PCR amplification or stored at −20 °C.

### PCR amplification

The DNA amplification reagents and extracted DNA retained in the supernatant after DNA extraction were brought to room temperature. The PCR reaction mixture was prepared with 10 µL of master mix, 5 µL of primer, and 0.8 µL of Taq enzyme per sample. Using PCR tandem tubes, 15 µL PCR reaction mixture and 5 µL extracted DNA were added to each tube. The following amplification program was run: 95 °C for 5 min, one cycle; 95 °C for 30 s, 58 °C for 30 s, 72 °C for 30 s, 5 cycles; 95 °C for 30 s, 55 °C foe 30 s, 72 °C for 30 s, 35 cycles; 72 °C for 3 min, one cycle; 4 °C for storage until analysis.

### Hybridization and detection

Microsphere hybridization solution (22 µL) and PCR amplification product (3 µL) were added to each well of a 96-well plate. Contents in each well were mixed and sealed. The hybridization program was conducted as follows: the first step was at 95 °C for 5 min, the second step was at 48 °C for 30 min, the third step was at 48 °C for 15 min, and the fourth step was at 48 °C until testing. At the end of the second step, the wells were opened, and 75 µL of streptavidin-phycoerythrin was quickly added. After the third step, the 96-well plate was removed and placed on a preheated metal plate at 48 °C before testing with the Luminex 200.

### Data analysis

The data were analyzed using the Tellgen Technology 3.2 software provided with the HPV assay kit.

### Treatment follow-up

According to personal information collected during physical examinations, women with positive HPV infection history (at least two years of HPV genotype assay results, including at least one year of positive infection history) in the Health Management Center of our hospital were selected. The following two groups were selected for telephonic follow-up: (1) women with positive infection history that were positive after their latest HPV genotype assay, and (2) women with positive infection history that had turned negative after their latest HPV genotype assay. Telephonic follow-up included discussion of spontaneous symptoms during HPV infection, treatment measures and prognosis, and ThinPrep cytology test (TCT) results or colposcopy reexamination.

### Statistical analysis

Statistical analysis was performed using Excel 2016 (Microsoft, Redmond, WA, USA) and SPSS 16.0 (SPSS, Inc., Chicago, IL, USA). The chi-squared test (*χ*^2^) was used to analyze qualitative data. The test level is *α* = 0.05, and *P* < 0.05 was considered statistically significant.

## Results

### Analysis of HPV infection and age distribution

This article analyzed 7,222 exfoliated cervical cell samples from 6,234 women divided into five groups by age (21–30, 31–40, 41–50, 51–60, >60 years). The age distribution of HPV infection is shown in Table 1. Among the women, 1068 had HPV infections, for a positive rate of 17.13% (1068/6234). There were statistically significant differences between HPV infection rates in different age groups, including the 21–30 and 31–40 (*P* = 0.002), 21–30 and 41–50 (*P* = 0.0003), 21–30 and 51–60 (*P* = 0.00003), and 51–60 and >60 age groups (*P* = 0.046), as determined by a paired *χ*^2^ test of the five groups; there were no other statistically significant differences between groups. As age increased, the infection rate increased until 60 years of age. The highest infection rate was in the 51–60-year age group (19.82%, 203/1024). The number of high-risk HPV infections was 977 (the number of infections was calculated according to HPV genotype, with multiple genotypes present in a single patient counted as multiple infections). The rate of high-risk HPV infection was 15.67% (977/6234), and the rate of low-risk infection 7.20% (449/6234). There were no significant differences between rates of high-risk and low-risk infections in different age groups (*P* = 0.520). As shown in Table 2, there were no statistically significant differences between numbers of single and multiple infections among the age groups (*P* = 0.075). HPV infection was dominated by single infection, with a rate of 13.01% (811/6234). Quintuple infection was the rarest, with a rate of 0.08% (5/6234).

**Table 1:** The age distribution of HPV infection.

Age group (years)	Positive n(%)^a^	Negative n(%)^a^	High-risk infection n(%)^a^	Low-risk infection n(%)^a^	Total
21–30^b^	105 (12.56)	731 (87.44)	105 (12.56)	41 (4.90)	836
31–40	340 (17.12)	1646 (82.88)	320 (16.11)	133 (6.70)	1986
41–50	382 (17.98)	1743 (82.02)	328 (15.44)	160 (7.53)	2125
51–60	203 (19.82)	821 (80.18)	189 (18.46)	99 (9.67)	1024
>60	38 (14.45)	225 (85.55)	35 (13.31)	16 (6.08)	263
Total	1068 (17.13)	5166 (82.87)	977 (15.67)	449 (7.20)	6234

**Notes.**

a The composition ratio (n(%)) is the ratio of women with positive infection to the total number of women in the age group.

b One patient was 18 years old.

**Table 2:** Age distribution of single and multiple infection in women with HPV infection.

Age group (years)	Single n(%)^a^	Double n(%)^a^	Triple n(%)^a^	Quadruple n(%)^a^	Quintet n(%)^a^	Total
21–30^b^	80 (9.57)	20 (2.39)	4 (0.48)	1 (0.12)	2 (0.24)	107
31–40	258 (12.99)	59 (2.97)	21 (1.06)	1 (0.05)	1 (0.05)	340
41–50	299 (14.07)	64 (3.01)	15 (0.71)	2 (0.09)	0 (0.00)	380
51–60	142 (13.87)	45 (4.39)	12 (1.17)	3 (0.29)	1 (0.10)	203
>60	32 (12.17)	2 (0.76)	2 (0.76)	1 (0.38)	1 (0.38)	38
Total	811 (13.01)	190 (3.05)	54 (0.87)	8 (0.13)	5 (0.08)	1068

**Notes.**

a The composition ratio (n(%)) is the ratio of women with positive infection to the total number of women in the age group.

b One patient was 18 years old.

### Distribution of HPV genotypes

The 27-genotype HPV assay was routinely employed in examinations of the 6234 women. The distribution of results for each genotype is shown in Table 3. The high-risk HPV types, especially HPV 52, 16, 53, 58, and 66, had proportionally high infection rates. Among the high-risk types, HPV 52 was detected at a rate of 2.42% (151/6234), and HPV 16 was detected at a rate of 2.01% (125/6234). Among low-risk HPV types, HPV 61, 81, 43, 55, and 44 had proportionally high infection rates. HPV 61 was the most commonly observed type, with a rate of 1.51% (94/6234). HPV 81 was the next most commonly observed type, with a rate of 1.41% (88/6234).

**Table 3:** Distribution of HPV genotypes in women who infected with HPV.

Type	HPV genotype	Frequency^a^	Positive ratio (%)^b^
High-risk	HPV52	151	2.42
	HPV16	125	2.01
	HPV53	89	1.43
	HPV58	82	1.32
	HPV66	63	1.01
	HPV45	61	0.98
	HPV39	57	0.91
	HPV51	56	0.90
	HPV18	52	0.83
	HPV56	49	0.79
	HPV59	45	0.72
	HPV68	42	0.67
	HPV33	32	0.51
	HPV35	27	0.43
	HPV31	22	0.35
	HPV82	22	0.35
	HPV26	2	0.03
Low-risk	HPV61	94	1.51
	HPV81	88	1.41
	HPV43	63	1.01
	HPV55	60	0.96
	HPV44	45	0.72
	HPV42	36	0.58
	HPV6	23	0.37
	HPV40	16	0.26
	HPV83	14	0.22
	HPV11	10	0.16

**Notes.**

a Women with multiple infections are counted as multiple times, so the total frequency is greater than the number of women infected with HPV.

b Positive rate is the ratio of women positive for a genotype to total participants.

### Follow-up results

Follow-up was conducted for 94 women who received multiple HPV tests and had a history of positive infections, 25 of which were lost to or refused follow-up, and 69 of which were assessed successfully. The history of positive HPV infection among the follow-up cases in women is shown in Table 4. Among them, 18 had a history of positive infection for 1–2 consecutive years, seven of which turned negative within 2 years, and 11 turned negative within 3 years. The remaining 51 women had a history of positive infection lasting 1–4 years that did not turn negative. The age distribution in respondents is shown in Table 5. Of the 18 women whose infection status turned negative, seven received treatment (one case of surgical (cervical cone resection), 6 cases of drug treatment), and 11 were untreated. Of the 51 patients whose status did not turn negative, 17 received treatment (five cases of surgical, 12 cases of drug treatment). The distribution of high-risk and low-risk infections among the followed-up women is shown in Table 6. High-risk HPV infections (56) were more common than low-risk HPV infections (13). Among the 51 women whose status did not turn negative, 19 had their TCT reexamined. Of the 19 women, 17 had normal TCT results, with no precancerous lesions found, and two had atypical squamous cells. Six women had colposcopies reexamined, five of which showed normal results, and one of which showed chronic cervical mucosal inflammation.

**Table 4:** HPV infection history of respondents.

Positive infection history	Turn to negative	Still positive	Total
One year	7	6	13
Two consecutive years	11	32	43
Three consecutive years	0	11	11
Four consecutive years	0	2	2

**Table 5:** Age distribution of HPV infection in respondents.

Age group (years)	Turn to negative		Still positive		Total
	Treated	Untreated	Treated	Untreated	
21–30	2	0	0	2	4
31–40	1	6	6	10	23
41–50	2	2	6	15	25
51–60	2	3	3	5	13
>60	0	0	2	2	4

**Table 6:** Distribution of high-risk and low-risk HPV infection in respondents.

HPV genotype	Turn to negative		Still positive		Total
	Treated	Untreated	Treated	Untreated	
High-risk	7	7	15	27	56
Low-risk	0	4	2	7	13

## Discussion

Hefei is located in eastern China, on the western end of the Yangtze River delta. It is the capital city of Anhui Province, with a large population (the total population is 7.965 million, including 3.908 million women). Screening and prevention of HPV-related diseases are vital to women’s health and economic development in Hefei.

Globally, the rate of HPV infection among women without cervical lesions is 11.6%–11.7%, with the highest rates in sub-Saharan Africa (24%), Eastern Europe (21.4%), and Latin America (16.1%) (Bruni et al., 2010; Forman et al., 2012). This study analyzed the HPV genotype assay results of 7,222 samples from 6,234 women undergoing routine physical examinations in Hefei. It was found that the total positive rate of HPV infection was 17.13%, which was higher than the average worldwide rate of HPV infection. All participants in our study were over 20 years old. Samples were obtained through routine physical examinations conducted by companies or organizations and did not include samples from women in hospitals or outpatients. Meanwhile, factors such as the level of economic development, population composition, city size, and medical conditions have also caused regional differences in HPV infection rates. Therefore, the HPV infection rate was lower than that in places like Beijing (25.2%) (Hong et al., 2014), Shanghai (18.98%) (Zhang et al., 2018), and Jiangsu Province (26.92%) (Zhang et al., 2019).

The prevalence of HPV infection in 37 cities in China shows a “two-peak” pattern, with one peak in the 15–19-year age group, followed by a second peak in the 50–60-year age group (Wang et al., 2015). The risk of HPV infection is highest after the first sexual encounter, and most women are able to clear the virus within six months to two years through the autoimmune system (Baseman & Koutsky, 2005). The HPV infection rate was reduced in women >20 years old, which might be related to their lifestyle, safety measures, and frequency of sexual intercourse. In our study, women were more susceptible at 51–60 years of age, suggesting that hormonal changes due to the decreases in ovarian function might lead to endocrine disorders, lower immune function, decreased ability to clear viruses, or the reactivation of the latent viruses (Althoff et al., 2009; Li, 2018).

The five most common high-risk HPV genotypes worldwide are HPV 16 (3.2%), HPV 18 (1.4%), HPV 52 (0.9%), HPV 31 (0.8%), and HPV 58 (0.7%) (Bruni et al., 2010). In mainland China, the five most prevalent high-risk genotypes are HPV 16, HPV 52, HPV 58, HPV 53, and HPV 18 (Li et al., 2019). Difference in the dominant high-risk genotypes of HPV infection also exists among cities in China. In Beijing, the five most common high-risk genotypes are HPV 16, HPV 52, HPV 58, HPV 33, and HPV 53 (Hong et al., 2014); in Shanghai, the five most common high-risk genotypes are HPV 16, HPV 58, HPV 52, HPV 51, and HPV 68 (Zhang et al., 2018); and in Jiangsu, the five most common high-risk genotypes are HPV 52, HPV 16, HPV 58, HPV 53, and HPV 51 (Zhang et al., 2019). In our study, the major high-risk genotypes were HPV 52 (2.42%), HPV 16 (2.01%), HPV 53 (1.43%), HPV 58 (1.32%) and HPV 66 (1.01%). HPV 16, which is associated with cervical cancer, was found in our cohort and is prevalent in all regions. Meanwhile, HPV 18, which is among the top five most globally prevalent types and is found in mainland China, was not among the most prevalent genotypes in Beijing, Shanghai, Jiangsu, or in our own cohort, in which it was ranked as the ninth most common high-risk genotype.

The development of the HPV vaccine was a leap forward in the prevention of cervical cancer. The HPV vaccine, which has been available in the United States since 2006, has been shown to prevent genital warts as well as cervical, vulvar, vaginal, and anal cancer. The latest version of the 9-valent vaccine (HPV 6, 11, 16, 18, 31, 33, 45, 52, and 58) can prevent 90% of cervical cancer. It was initially recommended for women aged 9–26 years, but this recommendation was recently extended to women up to 45 years old (Huh et al., 2017). HPV 52 and HPV 16 were the main types of infection in this study. Compared with the bivalent vaccine (HPV 16 and 18) and the quadrivalent vaccine (HPV 6, 11, 16, and 18), the 9-valent vaccine is more suitable for women in Hefei. The development of regionally-specific vaccines, especially those that prevent infection by HPV 53, will be more conducive to the prevention of HPV infection in Hefei.

The present study represents the first collection of a large number of physical examination samples for the analysis of HPV infection in women in Hefei and follow-up of case in women with 1–4 years of continuous infection. However, it has the following limitations. First, the HPV genotype assay is usually not performed in women under 20 years of age due to invasive injuries caused by the collection of exfoliated cervical cells. Thus, this study lacks data for women under 20 years of age. Second, the specimens selected in this article are all from the Health Management Center, which examines relatively healthy people. Therefore, the HPV infection rate may be lower than the true population rate and may not present a full picture of the HPV infection of women in the region or country. Third, participants with HPV infections may change jobs or choose other hospitals for physical examinations, making follow-up difficult. Thus, a comprehensive analysis of every patient at our hospital was impossible in this study. In the future, we hope to expand the sample to the entire Hefei area to analyze HPV infection and prevention comprehensively.

## Conclusions

In summary, the results of this research suggested that the positive rate of HPV infection in women of Hefei was 17.13%, and the negative conversion rate in women with continuous positive infection was low. The most common high-risk HPV genotypes are HPV 52, 16, 53, 58 and 66, and HPV genotype assays combined with TCT and colposcopy can be used to confirm diagnoses if necessary. Early vaccination, early diagnosis, and early treatment measures are necessary to prevent the occurrence of cervical cancer and other HPV-related lesions.

##  Supplemental Information

10.7717/peerj.10179/supp-1 Supplemental Information 1 The raw specimen data on women infected with HPV 1068 women had HPV infections, including single infection and multiple infections.

10.7717/peerj.10179/supp-2 Supplemental Information 2 The results of chi-square test between HPV infection rates in different age groups There were statistically significant differences between HPV infection rates in different age groups, including the 21–30 and 31–40 (*P* = 0.002), 21–30 and 41–50 (*P* = 0.0003), 21–30 and 51–60 (*P* = 0.00003), and 51–60 and < 60 years groups (*P* = 0.046); there were no other statistically significant differences between groups.

10.7717/peerj.10179/supp-3 Supplemental Information 3 The information and results of telephone follow-up respondents Sixty-nine women that had a history of 1 to 4 years of positive infection were followed-up, and 18 [one surgical (cervical cone resection), 6 drug,11 untreated] had infections turn negative. Seventeen of the 51 women whose infections did not turn negative received treatment.

## Additional Information and Declarations

### Data Availability

The following information was supplied regarding data availability: The raw specimen data, chi-squared test results and the telephone follow-up respondents and results are available in the Supplemental Files.
