# Supplementary material for: Human papillomavirus infection and follow-up on positive results in 7222 female samples obtained from 2016 to 2019 in Hefei, China
Source: PeerJ. 2020 Oct 20;8:e10179. doi: 10.7717/peerj.10179 (PMC7583622; doi:10.7717/peerj.10179)
Supplement: Supplemental Information 2 — There were statistically significant differences between HPV infection rates in different age groups, including the 21–30 and 31–40 (P = 0.002), 21–30 and 41–50 (P = 0.0003), 21–30 and 51–60 (P = 0.00003), and 51–60 and < 60 years groups (P = 0.046); there were no other statistically significant differences between groups. [file peerj-08-10179-s002.pdf]

Crosstabs

[DataSet0]

Case Processing Summary

	Cases					
	Valid		Missing		Total	
	N	Percent	N	Percent	N	Percent
r * c	6234	100.0%	0	.0%	6234	100.0%

r * c Crosstabulation

			c		Total
			1	2	
r	1	Count	105	731	836
		% within r	12.6%	87.4%	100.0%
	2	Count	340	1646	1986
		% within r	17.1%	82.9%	100.0%
	3	Count	382	1743	2125
		% within r	18.0%	82.0%	100.0%
	4	Count	203	821	1024
		% within r	19.8%	80.2%	100.0%
	5	Count	38	225	263
		% within r	14.4%	85.6%	100.0%
Total		Count	1068	5166	6234
		% within r	17.1%	82.9%	100.0%

Chi-Square Tests

	Value	df	Asymp. Sig. (2-sided)
Pearson Chi-Square	19.940[a]	4	.001
Likelihood Ratio	20.800	4	.000
Linear-by-Linear Association	8.549	1	.003
N of Valid Cases	6234		

a. 0 cells (.0%) have expected count less than 5. The minimum expected count is 45.06.

Crosstabs

[DataSet0]

Case Processing Summary

	Cases					
	Valid		Missing		Total	
	N	Percent	N	Percent	N	Percent
r * c	1426	100.0%	0	.0%	1426	100.0%

r * c Crosstabulation

			c		Total
			1	2	
r	1	Count	105	41	146
		% within r	71.9%	28.1%	100.0%
	2	Count	320	133	453
		% within r	70.6%	29.4%	100.0%
	3	Count	328	160	488
		% within r	67.2%	32.8%	100.0%
	4	Count	189	99	288
		% within r	65.6%	34.4%	100.0%
	5	Count	35	16	51
		% within r	68.6%	31.4%	100.0%
Total		Count	977	449	1426
		% within r	68.5%	31.5%	100.0%

Chi-Square Tests

	Value	df	Asymp. Sig. (2-sided)
Pearson Chi-Square	3.231[a]	4	.520
Likelihood Ratio	3.239	4	.519
Linear-by-Linear Association	2.481	1	.115
N of Valid Cases	1426		

a. 0 cells (.0%) have expected count less than 5. The minimum expected count is 16.06.

Crosstabs

[DataSet0]

Case Processing Summary

	Cases					
	Valid		Missing		Total	
	N	Percent	N	Percent	N	Percent
R * C	2822	100.0%	0	.0%	2822	100.0%

R * C Crosstabulation

			C		Total
			1	2	
R	1	Count	105	731	836
		% within R	12.6%	87.4%	100.0%
	2	Count	340	1646	1986
		% within R	17.1%	82.9%	100.0%
Total		Count	445	2377	2822
		% within R	15.8%	84.2%	100.0%

Chi-Square Tests

	Value	df	Asymp. Sig. (2-sided)	Exact Sig. (2-sided)	Exact Sig. (1-sided)
Pearson Chi-Square	9.211[a]	1	.002		
Continuity Correction[b]	8.871	1	.003		
Likelihood Ratio	9.551	1	.002		
Fisher's Exact Test				.002	.001
Linear-by-Linear Association	9.207	1	.002		
N of Valid Cases[b]	2822				

a. 0 cells (.0%) have expected count less than 5. The minimum expected count is 131.83.

b. Computed only for a 2x2 table

Crosstabs

[DataSet0]

Case Processing Summary

	Cases					
	Valid		Missing		Total	
	N	Percent	N	Percent	N	Percent
R * C	2961	100.0%	0	.0%	2961	100.0%

R * C Crosstabulation

			C		Total
			1	2	
R	1	Count	105	731	836
		% within R	12.6%	87.4%	100.0%
	2	Count	382	1743	2125
		% within R	18.0%	82.0%	100.0%
Total		Count	487	2474	2961
		% within R	16.4%	83.6%	100.0%

Chi-Square Tests

	Value	df	Asymp. Sig. (2-sided)	Exact Sig. (2-sided)	Exact Sig. (1-sided)
Pearson Chi-Square	12.810[a]	1	.0003		
Continuity Correction[b]	12.419	1	.0004		
Likelihood Ratio	13.390	1	.0003		
Fisher's Exact Test				.0003	.0002
Linear-by-Linear Association	12.805	1	.0003		
N of Valid Cases[b]	2961				

a. 0 cells (.0%) have expected count less than 5. The minimum expected count is 137.50.

b. Computed only for a 2x2 table

Crosstabs

[DataSet0]

Case Processing Summary

	Cases					
	Valid		Missing		Total	
	N	Percent	N	Percent	N	Percent
R * C	1860	100.0%	0	.0%	1860	100.0%

R * C Crosstabulation

			C		Total
			1	2	
R	1	Count	105	731	836
		% within R	12.6%	87.4%	100.0%
	2	Count	203	821	1024
		% within R	19.8%	80.2%	100.0%
Total		Count	308	1552	1860
		% within R	16.6%	83.4%	100.0%

Chi-Square Tests

	Value	df	Asymp. Sig. (2-sided)	Exact Sig. (2-sided)	Exact Sig. (1-sided)
Pearson Chi-Square	17.578[a]	1	.00003		
Continuity Correction[b]	17.056	1	.00004		
Likelihood Ratio	17.918	1	.00002		
Fisher's Exact Test				.00003	.00002
Linear-by-Linear Association	17.569	1	.00003		
N of Valid Cases[b]	1860				

a. 0 cells (.0%) have expected count less than 5. The minimum expected count is 138.43.

b. Computed only for a 2x2 table

Crosstabs

[DataSet0]

Case Processing Summary

	Cases					
	Valid		Missing		Total	
	N	Percent	N	Percent	N	Percent
R * C	1099	100.0%	0	.0%	1099	100.0%

R * C Crosstabulation

			C		Total
			1	2	
R	1	Count	105	731	836
		% within R	12.6%	87.4%	100.0%
	2	Count	38	225	263
		% within R	14.4%	85.6%	100.0%
Total		Count	143	956	1099
		% within R	13.0%	87.0%	100.0%

Chi-Square Tests

	Value	df	Asymp. Sig. (2-sided)	Exact Sig. (2-sided)	Exact Sig. (1-sided)
Pearson Chi-Square	.631[a]	1	.427		
Continuity Correction[b]	.475	1	.491		
Likelihood Ratio	.618	1	.432		
Fisher's Exact Test				.462	.243
Linear-by-Linear Association	.630	1	.427		
N of Valid Cases[b]	1099				

a. 0 cells (.0%) have expected count less than 5. The minimum expected count is 34.22.

b. Computed only for a 2x2 table

Crosstabs

```
[DataSet0]
```

Case Processing Summary

	Cases					
	Valid		Missing		Total	
	N	Percent	N	Percent	N	Percent
R * C	4111	100.0%	0	.0%	4111	100.0%

R * C Crosstabulation

			C		Total
			1	2	
R	1	Count	340	1646	1986
		% within R	17.1%	82.9%	100.0%
	2	Count	382	1743	2125
		% within R	18.0%	82.0%	100.0%
Total		Count	722	3389	4111
		% within R	17.6%	82.4%	100.0%

Chi-Square Tests

	Value	df	Asymp. Sig. (2-sided)	Exact Sig. (2-sided)	Exact Sig. (1-sided)
Pearson Chi-Square	.520[a]	1	.471		
Continuity Correction[b]	.463	1	.496		
Likelihood Ratio	.521	1	.471		
Fisher's Exact Test				.486	.248
Linear-by-Linear Association	.520	1	.471		
N of Valid Cases[b]	4111				

a. 0 cells (.0%) have expected count less than 5. The minimum expected count is 348.79.

b. Computed only for a 2x2 table

Crosstabs

[DataSet0]

Case Processing Summary

	Cases					
	Valid		Missing		Total	
	N	Percent	N	Percent	N	Percent
R * C	3010	100.0%	0	.0%	3010	100.0%

R * C Crosstabulation

			C		Total
			1	2	
R	1	Count	340	1646	1986
		% within R	17.1%	82.9%	100.0%
	2	Count	203	821	1024
		% within R	19.8%	80.2%	100.0%
Total		Count	543	2467	3010
		% within R	18.0%	82.0%	100.0%

Chi-Square Tests

	Value	df	Asymp. Sig. (2-sided)	Exact Sig. (2-sided)	Exact Sig. (1-sided)
Pearson Chi-Square	3.342[a]	1	.068		
Continuity Correction[b]	3.162	1	.075		
Likelihood Ratio	3.304	1	.069		
Fisher's Exact Test				.072	.038
Linear-by-Linear Association	3.341	1	.068		
N of Valid Cases[b]	3010				

a. 0 cells (.0%) have expected count less than 5. The minimum expected count is 184.73.

b. Computed only for a 2x2 table

Crosstabs

[DataSet0]

Case Processing Summary

	Cases					
	Valid		Missing		Total	
	N	Percent	N	Percent	N	Percent
R * C	2249	100.0%	0	.0%	2249	100.0%

R * C Crosstabulation

			C		Total
			1	2	
R	1	Count	340	1646	1986
		% within R	17.1%	82.9%	100.0%
	2	Count	38	225	263
		% within R	14.4%	85.6%	100.0%
Total		Count	378	1871	2249
		% within R	16.8%	83.2%	100.0%

Chi-Square Tests

	Value	df	Asymp. Sig. (2-sided)	Exact Sig. (2-sided)	Exact Sig. (1-sided)
Pearson Chi-Square	1.185[a]	1	.276		
Continuity Correction[b]	1.002	1	.317		
Likelihood Ratio	1.227	1	.268		
Fisher's Exact Test				.294	.158
Linear-by-Linear Association	1.185	1	.276		
N of Valid Cases[b]	2249				

a. 0 cells (.0%) have expected count less than 5. The minimum expected count is 44.20.

b. Computed only for a 2x2 table

Crosstabs

[DataSet0]

Case Processing Summary

	Cases					
	Valid		Missing		Total	
	N	Percent	N	Percent	N	Percent
R * C	3149	100.0%	0	.0%	3149	100.0%

R * C Crosstabulation

			C		Total
			1	2	
R	1	Count	382	1743	2125
		% within R	18.0%	82.0%	100.0%
	2	Count	203	821	1024
		% within R	19.8%	80.2%	100.0%
Total		Count	585	2564	3149
		% within R	18.6%	81.4%	100.0%

Chi-Square Tests

	Value	df	Asymp. Sig. (2-sided)	Exact Sig. (2-sided)	Exact Sig. (1-sided)
Pearson Chi-Square	1.560[a]	1	.212		
Continuity Correction[b]	1.440	1	.230		
Likelihood Ratio	1.546	1	.214		
Fisher's Exact Test				.221	.115
Linear-by-Linear Association	1.559	1	.212		
N of Valid Cases[b]	3149				

a. 0 cells (.0%) have expected count less than 5. The minimum expected count is 190.23.

b. Computed only for a 2x2 table

Crosstabs

[DataSet0]

Case Processing Summary

	Cases					
	Valid		Missing		Total	
	N	Percent	N	Percent	N	Percent
R * C	2388	100.0%	0	.0%	2388	100.0%

R * C Crosstabulation

			C		Total
			1	2	
R	1	Count	382	1743	2125
		% within R	18.0%	82.0%	100.0%
	2	Count	38	225	263
		% within R	14.4%	85.6%	100.0%
Total		Count	420	1968	2388
		% within R	17.6%	82.4%	100.0%

Chi-Square Tests

	Value	df	Asymp. Sig. (2-sided)	Exact Sig. (2-sided)	Exact Sig. (1-sided)
Pearson Chi-Square	2.009[a]	1	.156		
Continuity Correction[b]	1.773	1	.183		
Likelihood Ratio	2.101	1	.147		
Fisher's Exact Test				.170	.089
Linear-by-Linear Association	2.009	1	.156		
N of Valid Cases[b]	2388				

a. 0 cells (.0%) have expected count less than 5. The minimum expected count is 46.26.

b. Computed only for a 2x2 table

Crosstabs

[DataSet0]

Case Processing Summary

	Cases					
	Valid		Missing		Total	
	N	Percent	N	Percent	N	Percent
R * C	1287	100.0%	0	.0%	1287	100.0%

R * C Crosstabulation

			C		Total
			1	2	
R	1	Count	203	821	1024
		% within R	19.8%	80.2%	100.0%
	2	Count	38	225	263
		% within R	14.4%	85.6%	100.0%
Total		Count	241	1046	1287
		% within R	18.7%	81.3%	100.0%

Chi-Square Tests

	Value	df	Asymp. Sig. (2-sided)	Exact Sig. (2-sided)	Exact Sig. (1-sided)
Pearson Chi-Square	3.973[a]	1	.046		
Continuity Correction[b]	3.628	1	.057		
Likelihood Ratio	4.173	1	.041		
Fisher's Exact Test				.051	.026
Linear-by-Linear Association	3.970	1	.046		
N of Valid Cases[b]	1287				

a. 0 cells (.0%) have expected count less than 5. The minimum expected count is 49.25.

b. Computed only for a 2x2 table

Crosstabs

```
[DataSet0]
```

Case Processing Summary

	Cases					
	Valid		Missing		Total	
	N	Percent	N	Percent	N	Percent
r * c	1068	100.0%	0	.0%	1068	100.0%

r * c Crosstabulation

			c					Total
			1	2	3	4	5	
r	1	Count	80	20	4	1	2	107
		% within r	74.8%	18.7%	3.7%	.9%	1.9%	100.0%
	2	Count	258	59	21	1	1	340
		% within r	75.9%	17.4%	6.2%	.3%	.3%	100.0%
	3	Count	299	64	15	2	0	380
		% within r	78.7%	16.8%	3.9%	.5%	.0%	100.0%
	4	Count	142	45	12	3	1	203
		% within r	70.0%	22.2%	5.9%	1.5%	.5%	100.0%
	5	Count	32	2	2	1	1	38
		% within r	84.2%	5.3%	5.3%	2.6%	2.6%	100.0%
Total		Count	811	190	54	8	5	1068
		% within r	75.9%	17.8%	5.1%	.7%	.5%	100.0%

Chi-Square Tests

	Value	df	Asymp. Sig. (2-sided)
Pearson Chi-Square	24.710[a]	16	.075
Likelihood Ratio	23.051	16	.112
Linear-by-Linear Association	.229	1	.632
N of Valid Cases	1068		

a. 11 cells (44.0%) have expected count less than 5. The minimum expected count is .18.
